# Cloning and codon optimization of a novel feline interferon omega gene for production by *Pichia pastoris* and its antiviral efficacy in polyethylene glycol-modified form

**DOI:** 10.1080/21505594.2022.2029330

**Published:** 2022-01-24

**Authors:** Yixin Wang, Sheng Jiang, Xiaoxia Jiang, Xiaobo Sun, Xueting Guan, Yanyan Han, Linhan Zhong, Houhui Song, Yigang Xu

**Affiliations:** a Key Laboratory of Applied Technology on Green-eco-healthy Animal Husbandry of Zhejiang Province, College of Animal Science & Technology College of Veterinary Medicine, Zhejiang A&f University, Hangzhou, P.R. China; b College of Animal Science & Technology, Northeast Agricultural University, Harbin, P.R. China; c Zhejiang Provincial Engineering Laboratory for Animal Health Inspection and Internet Technology, College of Animal Science & Technology College of Veterinary Medicine, Zhejiang A&f University, Hangzhou, P.R. China; d Zhejiang International Science and Technology Cooperation Base for Veterinary Medicine and Health Management, College of Animal Science & Technology College of Veterinary Medicine, Zhejiang A&f University, Hangzhou, P.R. China

**Keywords:** feIFN-ω, codon optimization, *Pichia pastoris*, antiviral efficacy

## Abstract

Feline viral diseases, such as feline panleukopenia, feline infectious peritonitis, and feline coronaviral enteritis, seriously endanger the health of cats, and restrict the development of pet industry. Meanwhile, there is a current lack of effective vaccines to protect against feline viral diseases. Thus, effective therapeutic agents are highly desirable. Interferons (IFNs) are important mediators of the antiviral host defense in animals, particularly type I IFNs. In this study, a novel feline IFN omega (feIFN-ω) gene was extracted from the cat stimulated with feline parvovirus (FPV) combined with poly(I:C), and following codon optimization encoding the feIFN-ω, the desired gene (feIFN-ω’) fragment was inserted into plasmid pPICZαA, and transformed into *Pichia pastoris* GS115, generating a recombinant *P. pastoris* GS115 strain expressing the feIFN-ω’. After induction, we found that the expression level of the feIFN-ω’ was two times more than that of feIFN-ω (*p* < 0.01). Subsequently, the feIFN-ω’ was purified and modified with polyethylene glycol, and its antiviral efficacy was evaluated *in vitro* and *in vivo*, using vesicular stomatitis virus (VSV) and FPV as model virus. Our results clearly demonstrated that the feIFN-ω’ had significant antiviral activities on both homologous and heterologous animal cells *in vitro*. Importantly, the feIFN-ω’ can effectively promote the expression of antiviral proteins IFIT3, ISG15, Mx1, and ISG56, and further enhance host defense to eliminate FPV infection *in vivo*, suggesting a potential candidate for the development of therapeutic agent against feline viral diseases.

## Introduction

Interferon (IFN) is an antiviral protein found from influenza virus-infected chicken embryos in 1957, and subsequent studies have demonstrated that human and animal cells also can produce IFNs, which are considered as the main natural immune barrier for the host against viral infections [1–5]. IFNs are the main choice of antiviral drugs in the current clinical use. According to the structure, functional characteristics, and receptor of IFNs, IFNs can be divided into three subtypes including type I, type II and type III [6]. Among them, type I IFNs play an important role in promoting host defense against virus infections, including IFN-α, IFN-β, IFN-ε, IFN-ω, IFN-κ, IFN-δ, IFN-τ, and IFN-ζ, in particular IFN-α, and IFN-ω, with potent immunomodulatory, antiviral, and antiproliferative properties [7]. Type II IFN, also known as IFN-γ, can induce a series of immune responses and regulate immune system, which acts as an antiviral agent by mainly inhibiting viral activity [8]. Type III IFN, or IFN-λ family, is composed of IFN-λ1, IFN-λ2, IFN-λ3, and IFN-λ4, with antiviral activity [9,10].

Nowadays, it is more and more common to keep companion animals, especially pet cats. However, cat viral diseases, such as feline infectious peritonitis caused by feline infectious peritonitis virus (FIPV), feline panleukopenia caused by feline parvovirus (FPV), and cat enteritis caused by feline enteric coronavirus seriously endanger the health of cats and the development of the pet cat industry [11–14]. In addition, there is a current lack of effective vaccines to protect against cat viral diseases. Therefore, effective therapeutic agents for cat viral diseases are highly desirable. IFN-ω, first reported in 1985 [15], is secreted primarily by leukocytes, which has now been found in humans [16] and some animals including cats [17,18], pigs [19], and horses [20], etc. IFN-ω combines with IFN receptor complex and activates phosphatidylinositol-3-kinase/protein kinase B (P13K/Akt) signal pathway to exert antiviral activity, thereby achieving the effect of inhibiting viruses [21,22]. Compared with IFN-α, IFN-ω can significantly inhibit virus replication [23–25], suggesting a promising antiviral agent.

In this study, in order to develop a potent antiviral agent for the treatment of cat viral diseases, a gene encoding a novel feline IFN-ω (feIFN-ω) was obtained from the peripheral blood of the cat stimulated with FPV combined with poly(I:C). Following codon optimization of the gene encoding feIFN-ω, the desired feIFN-ω’ was produced by a recombinant *Pichia pastoris* strain, and after being modified with polyethylene glycol, the antiviral efficacy of the feIFN-ω’ was evaluated *in vitro* and *in vivo*.

## Materials and methods

### Animal, viruses, bacterial strain, cells, and plasmid

Healthy 4-month-old Dragon Li cats (*n* = 20) were purchased from a pet market in China. Animal experiments were carried out in accordance with the recommendations in the Guide for the Care and Use of Laboratory Animals of the National Institutes of Health, and were approved by the Ethical Committee for Animal Experiments of Zhejiang A&F University (ZAFUAC2021021), China. Vesicular stomatitis virus (VSV) and feline parvovirus (FPV) were kept in our laboratory. *Pichia pastoris* strain GS115 (*P. pastoris* GS115) kept in our laboratory was cultured in Yeast-extract Peptone Dextrose (YPD) medium (Solarbio, China) at 30°C. Madin–Darby bovine kidney (MDBK) cells, Madin–Darby canine kidney (MDCK) cells, and feline kidney (F81) cells were cultured in Dulbecco’s Modified Eagle Medium, DMEM (Gibco, USA) supplemented with 10% fetal bovine serum, FBS (Gibco, USA) in a 5% CO_2_ incubator at 37°C. The plasmid pPICZαA kept in our laboratory was used to construct recombinant *P. pastoris* expressing feline IFN-ω.

### Cloning of gene encoding feIFN-ω

The cat was co-stimulated by 500 µL of FPV (10^3^ TCID_50_) and 500 µL of 1.0 mg/mL poly(I:C) (Sigma, USA) through subcutaneous injection route for three consecutive days. On day 10 post-stimulation, the peripheral blood of the cat was collected, and the total RNA was extracted by Total RNA Isolation kit (Invitrogen, USA) followed by reverse transcription, generating first-strand cDNA. Next, using the cDNA as template, a gene encoding feIFN-ω was obtained by PCR amplification with the primer pair listed in Table 1. After that, the PCR product of interest was purified and subcloned into pMD-19 T plasmid, generating recombinant plasmid pMD-ω, followed by gene sequencing (Kumei, China).

**Table 1.** Primers used in this study

Gene	Primer sequences (5’→3’)	References
feIFN-ω	F: ATGGCCCTCCTGCTCCC	In this work
	R: AGATGAGCCCAGGTCTCCAT	
FPV-VP2	F: CTGGAGGACGAGGGATACAGTGAC	In this work
	R: GGTCGCCGAGGAGGACAAGG	
Mx1	F: TTCGGAGGTGGAGGAGGCAATC	In this work
	R: CAGGGAGGTCTATCAGGGTCAGATC	
ISG15	F: AACCACAAGGGTCGCACCATTG	In this work
	R: TGCTGGCATATCTGCTGCTTGAG	
IFIT3	F: TGAAGCTGGCAAGAATGGAGAGAAG	In this work
	R: GGAGGTCGGTGACATCAGAATATGC	
ISG56	F: GCAACTACGCCTGGCTGTATCAC	In this work
	R: CCCACCCTTCCTCACAGTCCATC	
IL-1β	F: ATTGTGGCTATGGAGAAACTGAAG	[38]
	R: TCTTCTTCAAAGATGCAGCAAAAG	
IL-4	F: CCCCTAAGAACACAAGTGACAAG	[42]
	R: CCTTTGAGGAATTTGGTGGAG	
IL-6	F: GTGTGACAACTATAACAAATGTGAGG	[38]
	R: GTCTCCTGATTGAACCCAGATTG	
IL-10	F: ACTTTCTTTCAAACCAAGGACGAG	[38]
	R: GGCATCACCTCCTCCAAATAAAAC	
IL-12	F: TGGCCTTCTGAAGCGTGTTG	[42]
	R: GAAGTACACAGTGGAGTGTCAGG	
TNF-α	F: TGCTTGTGCCTCAGCCTC	In this work
	R: ACTGGCTTGTCACTCGGAGT	
β-actin	F: GACTACCTCATGAAGATCCTCACG	[38]
	R: CCTTGATGTCACGCACAATTTCC	

### Gene sequence analysis

The gene encoding feIFN-ω were deposited in GenBank under accession number MT754935. Homology and phylogenetic tree analysis of the feIFN-ω gene were performed using DNASTAR and MEGA7 software.

### Codon optimization of the feIFN-ω gene sequences

In this study, we aimed to produce the feIFN-ω by yeast to test its antiviral efficacy *in vitro* and *in vivo*, and therefore we optimized the codons of the gene encoding the feIFN-ω according to the codon usage bias of *P. pastoris*, including predominant codons usage, the adjustment of GC content, and AT-rich repeat region. However, no amino acid sequences of the feIFN-ω were changed. Subsequently, the optimized gene (named feIFN-ω’) was synthesized (Kumei, China), which was harbored in a recombinant plasmid pMD-ω’.

### Expression of the feIFN-ω*’* by recombinant P. pastoris and modification with polyethylene glycol

The recombinant plasmid pMD-ω containing the feIFN-ω gene (or pMD-ω’ containing the optimized feIFN-ω’ gene) and vector pPICZαA were, respectively, digested by *Bam*H I and *Kpn* I (NEB, USA), and the gene fragments of interest were purified, followed by ligation with T4 DNA ligase (Takara, China), generating the recombinant plasmid pPICZαA-ω (or pPICZαA-ω’). Next, the recombinant plasmid pPICZαA-ω (or pPICZαA-ω’) was linearized by AVRII enzyme digestion (NEB, USA), and then the linearized pPICZαA-ω (or pPICZαA-ω’) was electroporated into the *P. pastoris* GS115 competent cells, followed by screening positive clones on YPD plates supplemented with 100 µg/mL of Zeocin (Sigma, USA), generating the recombinant strains named GS115-pPICZαA-ω or GS115-pPICZαA-ω’. In order to produce the feline interferon, the recombinant strain GS115-pPICZαA-ω (or GS115-pPICZαA-ω’) was grown overnight to an OD_600_ of approximately 1.5 in BMGY medium (Solarbio, China). After centrifugation, the cells pellets were transferred into BMMY medium containing 1.5% YNB, biotin, and 0.05% methanol (Solarbio, China), and cultured for 120 h. Sterile-filtered methanol was supplemented every 24 h to maintain induction condition. The expression level of the feIFN-ω in GS115-pPICZαA-ω (and GS115-pPICZαA-ω’) and its culture supernatants was analyzed by 12% SDS-PAGE. After that, the feIFN-ω’ was purified by Ni^2+^ affinity chromatography column, and PEGylation was conducted in sodium borate buffer with the feIFN-ω’ and 40 kDa mPEG-2-N-hydroxysuccinimide, and then the polyethylene glycol-modified feIFN-ω’ was purified by a Q-Sepharose column [26].

#### Determination of antiviral activity of the feIFN-ω’ in vitro

The antiviral activity of the recombinant feIFN-ω’ was determined by microdose cytopathogenic effect inhibition assay (MCIA) *in vitro* using VSV and FPV as model viruses. The supernatants from induced GS115-pPICZαA-ω’ were serially 10-fold diluted in DMEM supplemented with 5% FBS, and were then added into a 96-well plate (8 replicates per dilution) followed by addition of MDBK cells to a concentration of 5 × 10^4^ cells/well. Then, the MDBK cells were incubated at 37°C in a 5% CO2 incubator for 24 h. After discarding the supernatants, the MDBK cells were infected with VSV (or FPV) at an MOI of 1.0. When the cytopathic effect (CPE) reached 100% in virus control group (untreated with feIFN-ω’), the cells were stained with 0.1% (w/v) of crystal violet, and incubated at 37°C for 30 min. After elution with 50% ethanol-0.1% acetic acid, absorbance was measured at 595 nm. In parallel, the supernatants from GS115-pPICZαA were used as negative control. Subsequently, the antiviral activity of the feIFN-ω’ purified by Ni^2+^ affinity chromatography column at a concentration of 0.001 ng ~ 10 ng was determined by MCIA, using FPV as model virus and INTERCAT IFN (Toray Industries, Japan) as feline IFN positive control. The test was repeated five times. Furthermore, the species-specific antiviral activity was also determined by the ability of the feIFN-ω’ to inhibit the CPE of VSV on F81 cells, MDCK cells, and MDBK cells. In parallel, the INTERCAT IFN was used as feline IFN control. The assay was repeated three times (8 replicates each sample).

#### Determination of antiviral activity of the polyethylene glycol-modified feIFN-ω’ in vivo

In order to determine the antiviral efficacy of the polyethylene glycol-modified feIFN-ω’ *in vivo*, eighteen 4-month-old Dragon Li cats were randomly divided into three groups: FPV infection group (cat was infected with 10^5^ TCID_50_ FPV via subcutaneous and digestive routes; *n* = 6), feIFN-ω’ treatment group (cat was infected with FPV followed by treatment with the polyethylene glycol-modified feIFN-ω’ at a dose of 150 μg/0.5 mL, twice, 5 days apart; *n* = 6), and normal control group (mock group; *n* = 6). On days 5 and 15 after challenge, three cats were randomly selected from each group, respectively, and the viral loads of the FPV in blood, kidney, liver, spleen, intestine, and feces of the cats were determined using a SYBR Green I-based real-time quantitative RT-PCR (RT-qPCR) assay with the primer pair targeting the FPV VP2 gene (listed in Table 1). Meanwhile, the presence of viral antigen in the intestinal tract of the cats from each group was detected on day 10 after the primary feIFN-ω’ treatment by an immunohistochemistry (IHC) assay using a mouse anti-FPV VP2 polyclonal antibody (prepared in our laboratory; diluted at 1:100) as the primary antibody and HRP-conjugated goat anti-mouse IgG antibody (Abcam, USA; diluted at 1:1000) as the secondary antibody. Moreover, the cats in each group were bled on days 0, 5, 10, and 15 after FPV infection using the blood-diluting pipettes, followed by total white blood cell (WBC) counts with Fuchs-Rosenthal counting chambers. At the same time, using β-actin as internal control, the mRNA transcript levels of antiviral proteins Mx1, ISG15, ISG56, and IFIT3, and cytokines including IL-1β, TNF-α, IL-4, IL-6, IL-10, and IL-12 in the blood samples of the cats from each group were determined on day 10 after feIFN-ω’ treatment by RT-qPCR assay with the primer pairs listed in Table 1. In addition, the protein expression levels of the antiviral proteins Mx1, ISG15, ISG56, and IFIT3 were determined by Western blot using rabbit anti-Mx1/ISG15/ISG56/IFIT3 polyclonal antibody (ABclonal, USA; diluted at 1:1000) as the primary antibody and HRP-conjugated goat anti-rabbit IgG antibody (Abcam, USA; diluted at 1:2000) as the secondary antibody, respectively. After that, the immunoblot band was visualized using chemiluminescent substrate reagent (Thermo Fisher Scientific, USA).

### Statistical analysis

In this work, data were shown as mean ± standard error (SE) values, and Tukey’s multiple comparison tests and one-way analysis of variance (ANOVA) were used to analyze the differences among groups by GraphPad Prism V8.0 software.

## Results

### Cloning and phylogenetic analysis of feIFN-ω gene

**Figure 1.**
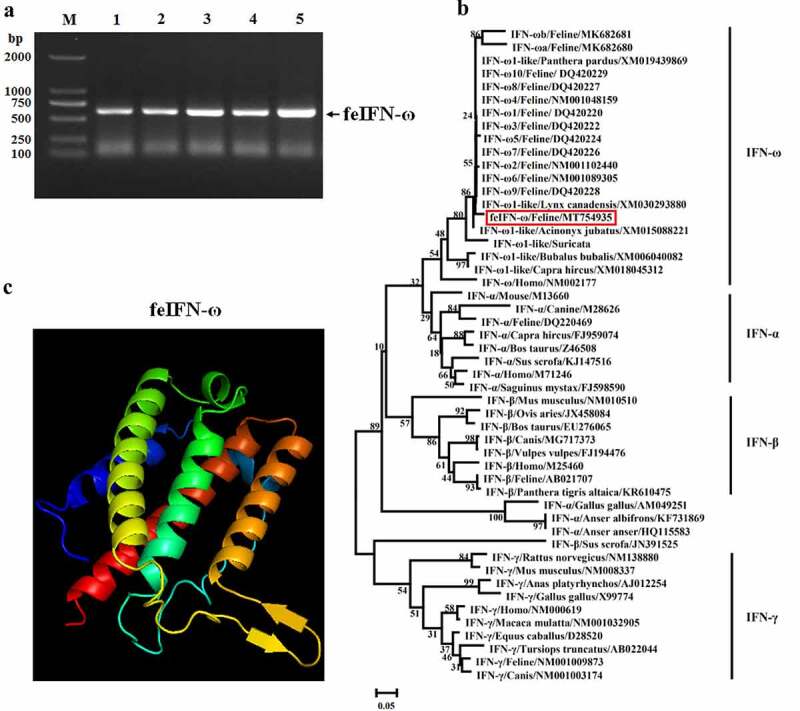
A: Amplification of feIFN-ω gene by RT-PCR. M: DNA Marker DL2000; lanes 1–5: the feIFN-ω gene amplified from the peripheral blood of cat. B: Phylogenetic tree analysis of the feIFN-ω gene by neighbor-joining method (1000 replicates) using MEGA 7 software. C: Predicted three-dimensional structure of the feIFN-ω.

After being stimulated by FPV combined with poly(I:C), a gene of approximately 612 bp encoding feIFN-ω was amplified by RT-PCR from the peripheral blood of the cat (Figure 1a), and phylogenetic analysis of the feIFN-ω gene was performed by the neighbor-joining method (1000 replicates) using MEGA 7 software to explore the evolutionary relationships between the feIFN-ω gene obtained in this study and other IFNs published in GenBank. As shown in Figure 1b, the feIFN-ω gene that was obtained in this study belonged to the type I IFN family, which shared relatively distant genetic relationships with other feIFN-ωs published in GenBank, indicating that a novel feIFN-ω was obtained in this study. We further predicted the three-dimensional (3D) structure of the feIFN-ω by an online tool (http://www.sbg.bio.ic.ac.uk), and result demonstrated that the 3D structure of the feIFN-ω conformed to the 3D structural characteristics of type I IFN (Figure 1c).

### Codon optimization of the gene encoding feIFN-ω and its expression by P. pastoris

**Figure 2.**
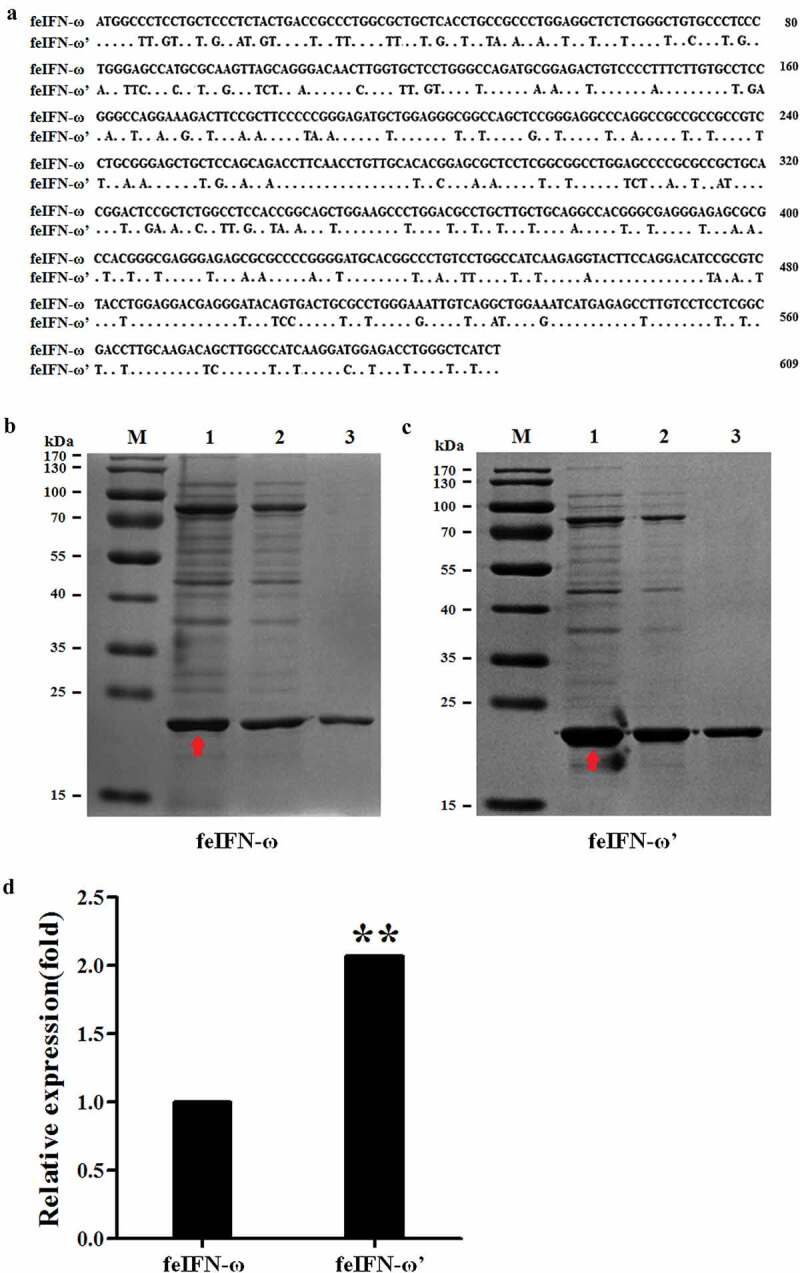
A: Codon optimization of the feIFN-ω according to the codon usage bias of *P. pastoris*. B: The expression level of feIFN-ω by the GS115-pPICZαA-ω before codon optimization. M: Protein marker; lane 1: feIFN-ω in cells; lane 2: feIFN-ω in supernatants; lane 3: purified feIFN-ω. C: The expression level of feIFN-ω’ by the GS115-pPICZαA-ω’ after codon optimization. M: Protein marker; lane 1: feIFN-ω’ in cells; lane 2: feIFN-ω’ in supernatants; lane 3: purified feIFN-ω’. D: Comparison of feIFN-ω expression levels before and after codon optimization.

In order to achieve highly efficient expression of the feIFN-ω by *P. pastoris*, the gene sequences encoding the feIFN-ω were optimized according to the codon usage bias of *P. pastoris* (Figure 2a). Subsequently, the recombinant strain GS115-pPICZαA-ω expressing feIFN-ω and strain GS115-pPICZαA-ω’ expressing feIFN-ω’ (optimized feIFN-ω) was constructed, respectively. Following induction with methanol, the IFN protein of interest that was expressed by the recombinant strains was detected by SDS-PAGE, and results showed that the feline IFN-ω can be effectively produced by the recombinant strain GS115-pPICZαA-ω (Figure 2b) and strain GS115-pPICZαA-ω’ (Figure 2c). By contrast, we found that the expression level of optimized feIFN-ω’ was two times more than that of unoptimized feIFN-ω (*p* < 0.01) (Figure 2d).

### Biological activity of the recombinant feIFN-ω’ in vitro

**Figure 3.**
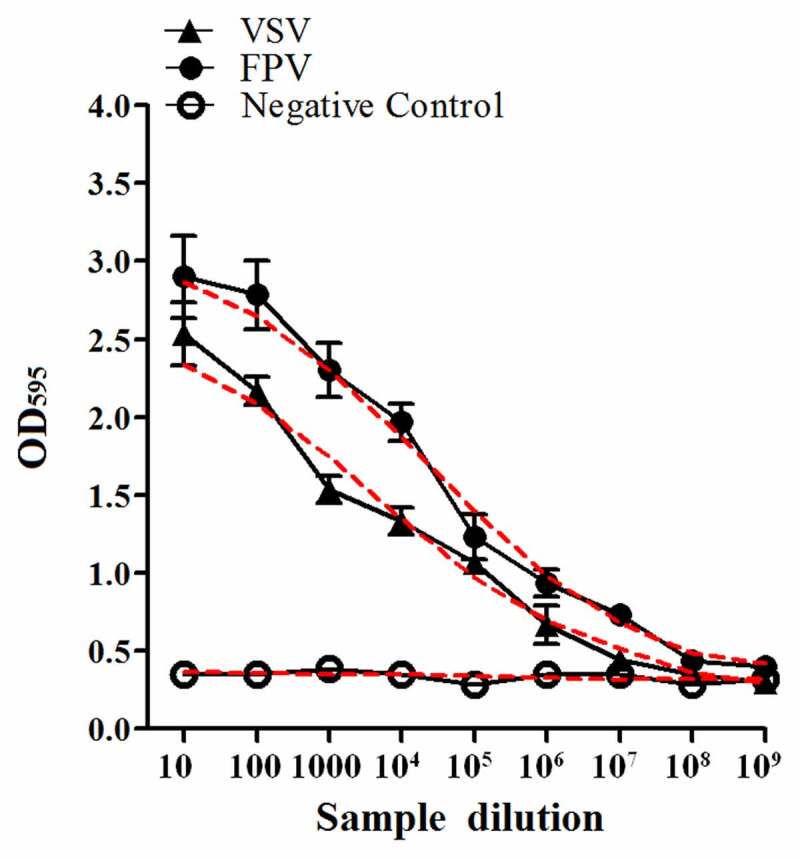
Antiviral activity of the supernatants of the GS115-pPICZαA-ω’ induced by methanol. F81 cells were incubated in the presence of the indicated dilutions of supernatants containing feIFN-ω for 24 h, followed by infection with VSV and FPV, respectively. When CPE in virus control group reached 100%, the cells were stained with crystal violet followed by measuring the absorbance at 595 nm. The connecting curve (red line in the figure) in each group was set up using GraphPad Prism 8.0 software.

**Figure 4.**
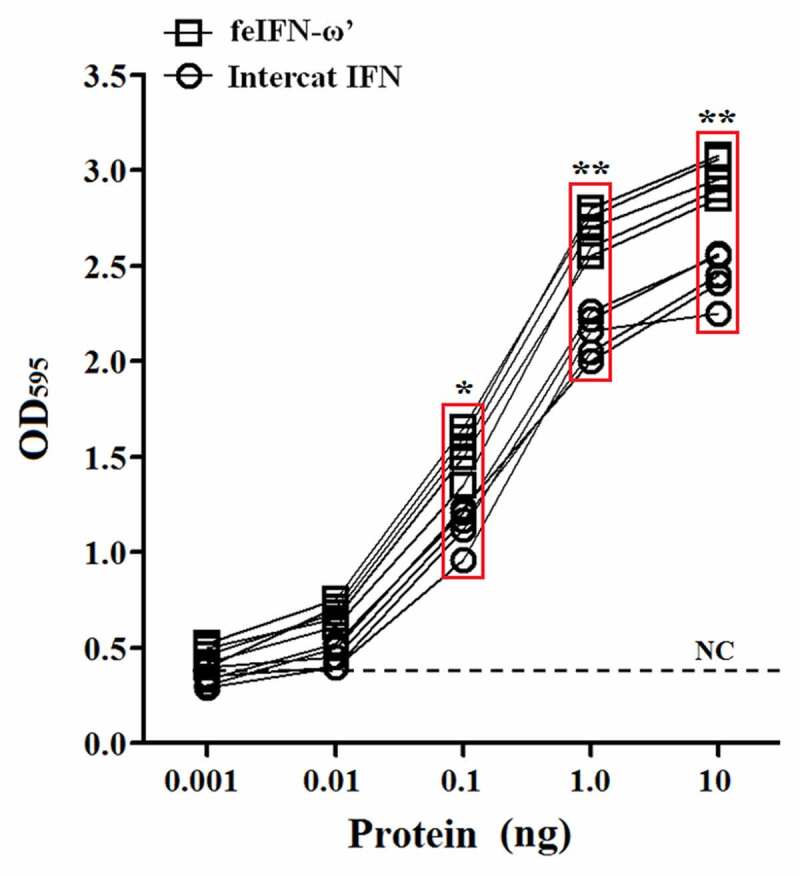
Antiviral activity of the purified feIFN-ω’ using FPV as model virus. F81 cells grown to confluence were treated with the indicated amounts of purified feIFN-ω’ for 24 h, followed by infection with FPV at an MOI of 1.0. When CPE in virus control group reached 100%, the cells were stained with crystal violet followed by measuring the absorbance at 595 nm. In parallel, Intercat IFN was used as positive control. *p < 0.05; **p < 0.01.

**Figure 5.**
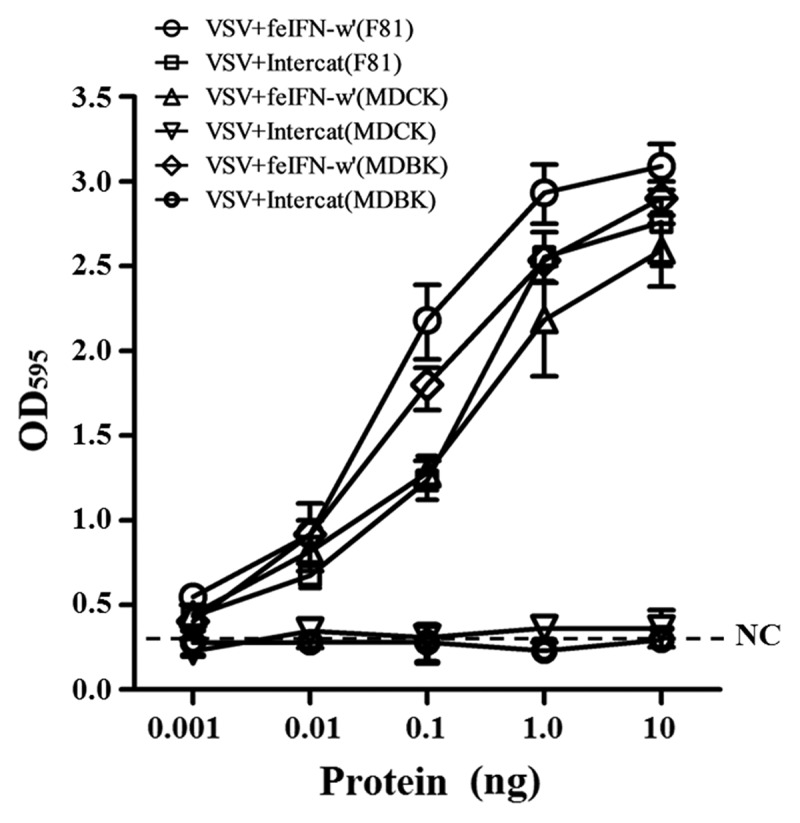
Antiviral activities of the purified feIFN-ω’ and Intercat IFN on F81 cells, MDCK cells, and MDBK cells utilizing a standard VSV assay.

In order to evaluate the biological activity of the recombinant GS115-expressed feIFN-ω’, we first tested the antiviral activity of the supernatants of the recombinant strain GS115-pPICZαA-ω’ induced with methanol, using VSV and FPV as model viruses. As shown in Figure 3, a significant reduction in the VSV- and FPV-induced CPE on the MDBK cells can be observed, exhibiting an effective antiviral activity with a dose-dependence. Subsequently, we tested the antiviral activity of the purified recombinant feIFN-ω’ expressed by the GS115-pPICZαA-ω’ using INTERCAT IFN as feline interferon-ω positive control, and results showed that the purified feIFN-ω’ displayed significant antiviral activity with a dose-dependence (Figure 4). By contrast, the antiviral activity of the recombinant feIFN-ω’ was better than that of the INTERCAT IFN control. Furthermore, we tested the species-specific antiviral activity of the purified feIFN-ω’ by determining the ability to inhibit the CPE of VSV on F81 cells, MDCK cells, and MDBK cells, using INTERCAT IFN as a control. As shown in Figure 5, a significant reduction in the VSV-induced CPE on the F81 cells, MDCK cells, and MDBK cells can be observed with a dose-dependence in the feIFN-ω’ treatment cell groups. However, in INTERCAT IFN treatment cell groups, significant reduction in the VSV-induced CPE on F81 cells can only be observed, but not on MDCK cells, and MDBK cells.

### Antiviral activity of the recombinant feIFN-ω’ in vivo

**Figure 6.**
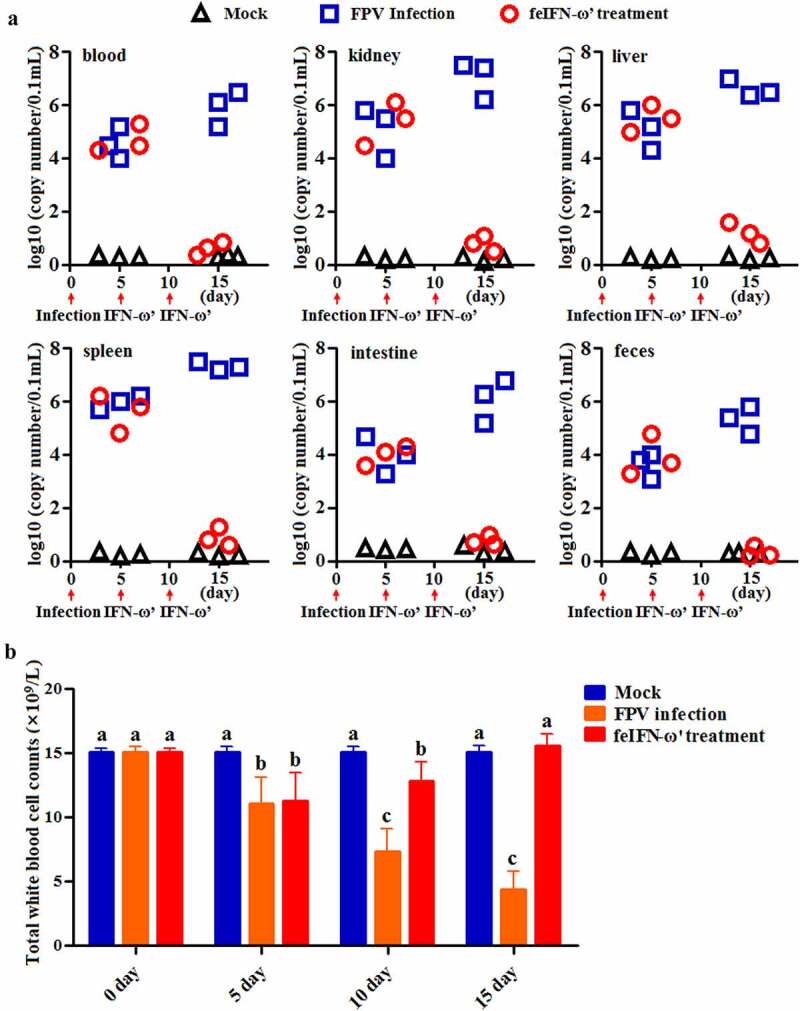
A: Changes of viral loads in blood, kidney, liver, spleen, intestine, and feces of the cats in normal control group, FPV infection group, and feIFN-ω treatment group determined by a RT-qPCR assay. B: The total WBC counts of the cats in each group.

In order to evaluate the antiviral activity of the recombinant feIFN-ω’ *in vivo*, the cats were infected with FPV followed by the feIFN-ω’ therapy. Our results showed that after the feIFN-ω’ treatment, virus loads in blood, kidney, liver, spleen, intestine, and feces of the cats in the feIFN-ω’ treatment group significantly decreased on days 10 after feIFN-ω’ treatment, indicating effective antiviral activity of the recombinant feIFN-ω’ *in vivo* (Figure 6a). However, in FPV-infected cat group (without feIFN-ω’ treatment), the virus titers in blood, kidney, liver, spleen, intestine, and feces of the cats significantly increased, and some cats developed severe clinical symptoms and were euthanized after the experiment. During a 15-day monitoring period, we counted the total WBC in blood of the cats in each group, and results showed that compared to the normal control group, the total WBC counts in FPV-infected cat group significantly decreased, while the total WBC counts of the FPV-infected cats were gradually returned to normal after treatment with the feIFN-ω’ (Figure 6b).

**Figure 7.**
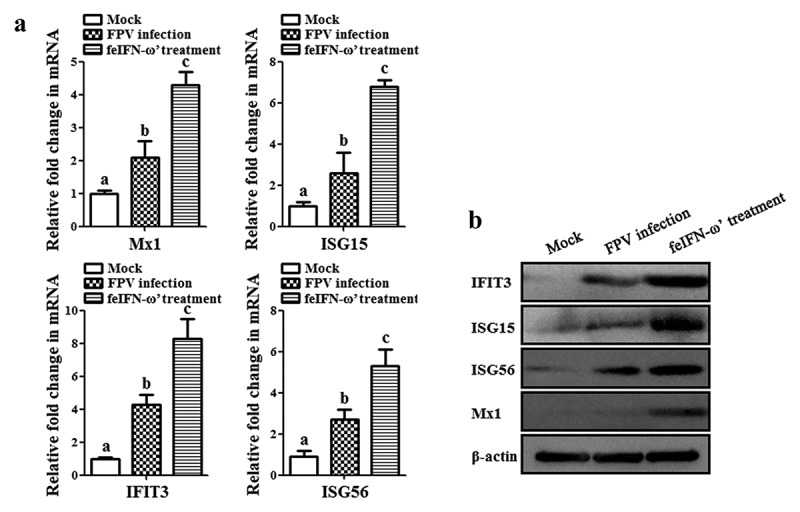
The mRNA transcript levels (a) and protein expression levels (b) of antiviral proteins Mx1, ISG15, ISG56, and IFIT3 in the blood of the cats from each group were detected by SYBR Green I RT-qPCR and Western blot, respectively, using β-actin as internal control. The lowercase letters “a versus b, and b versus c” indicate significant difference of *p* < 0.05; “a versus c” indicates significant difference of *p* < 0.01.

**Figure 8.**
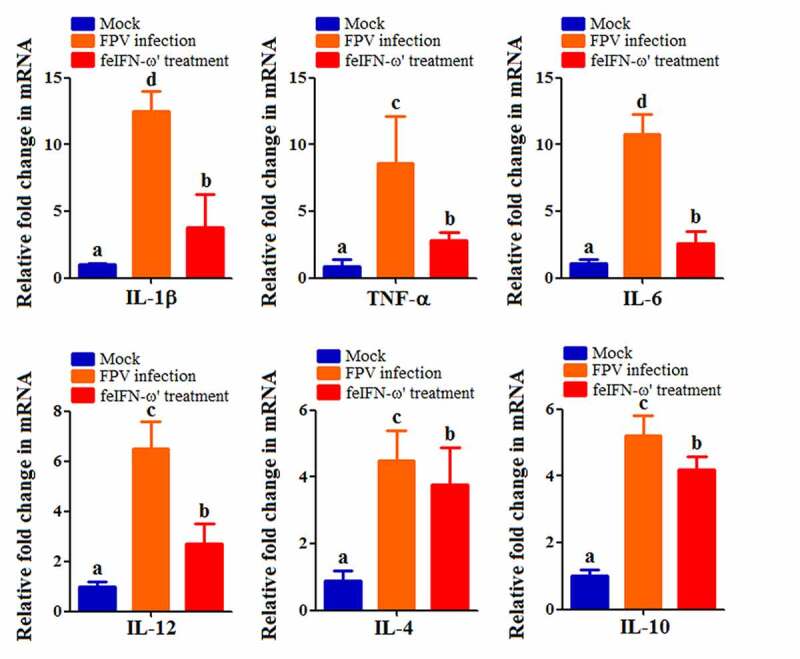
The mRNA transcript levels of cytokines IL-1β, TNF-α, IL-4, IL-6, IL-10, and IL-12 in the blood samples of the cats from each group were determined by RT-qPCR assay on day 10 after feIFN-ω’ treatment. The lowercase letters “a versus b, b versus c, and c versus d” indicate significant difference of *p* < 0.05; “a versus c, and b versus d” indicates significant difference of *p* < 0.01; “a versus d” indicates significant difference of *p* < 0.001.

**Figure 9.**
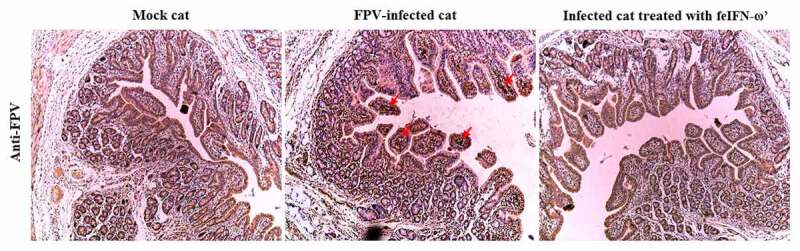
The presence of viral antigen in the intestinal tract of the cats from each group was detected on day 10 after feIFN-ω’ treatment by IHC assay using mouse anti-FPV VP2 polyclonal antibody as the primary antibody and HRP-conjugated goat anti-mouse IgG antibody as the secondary antibody.

In addition, on day 10 after the feIFN-ω’ treatment, we determined the mRNA transcript levels and protein expression levels of the antiviral proteins Mx1, ISG15, ISG56, and IFIT3 in the blood of the cats from each group using RT-qPCR assay and Western blot assay, respectively. As shown in Figure 7, both the mRNA transcript levels (Figure 7a) and protein expression levels (Figure 7b) of these antiviral proteins were significantly higher than those in mock control group (*p* < 0.01) and FPV infection group (*p* < 0.05), indicating that the feIFN-ω’ can effectively promote the expression of antiviral proteins. Furthermore, we also determined the mRNA transcript levels of cytokines IL-1β, TNF-α, IL-4, IL-6, IL-10, and IL-12 in the blood samples of the cats from each group by RT-qPCR, and results showed that compared to the FPV infection group, the levels of these cytokines significantly decreased (*p* < 0.05 or *p* < 0.01) in the feIFN-ω’ treatment, but still significantly higher (*p* < 0.05) than those in the mock control group (Figure 8). Finally, we used the IHC assay to detect the FPV loaded in the intestinal tract of the cat from each group on day 10 after feIFN-ω’ treatment, and found that there was no virus detected in the feIFN-ω’ treatment group, while large amounts of viruses were observed in the FPV infection group (Figure 9).

## Discussion

Viral diseases in pet cats such as feline leukemia, feline panleukopenia, and feline infectious peritonitis, with high incidences, are still difficult to prevent due to the lack of effective vaccine products. IFN with good antiviral activity shows positive effects for treating viral diseases in cats [27–30]. In this study, a gene encoding a novel feIFN-ω was cloned from the peripheral blood of cat stimulated with FPV combined with ploy (I:C), and with codon optimization of the feIFN-ω gene, the IFN-ω was produced by a recombinant *P. pastoris* induced with methanol, and its antiviral activity was evaluated *in vitro* and *in vivo*.

Protein expression system includes prokaryotic and eukaryotic systems. Although prokaryotic expression system is most commonly used [18], the activity of expressed proteins is not good. For example, using prokaryotic system to produce interferon, it is necessary to extract the inclusion bodies in cells to obtain the protein of interest, followed by denaturation, refolding, conformational change *in vitro*, which is time-consuming and laborious, increases the production cost, and also leads to serious protein loss with some biological activity loss [31,32]. Yeast expression system is a kind of eukaryotic expression system, and the expressed target protein can complete the modified folding closest to the natural protein in cells [33], and can be secreted into the supernatants, which is conducive to target protein extraction and purification, achieving the goal of low cost and high return [34]. Therefore, we used a yeast (*P. pastoris*) expression system to produce the feIFN-ω in this study, and our result showed that the feIFN-ω can be effectively expressed by *P. pastoris* and secreted into supernatants.

Importantly, the efficient expression of exogenous protein in *P. pastoris* is related to the conformity of expression system, selection of vectors, expression conditions, and other factors [35,36]. Moreover, rare codons will affect the transcription and translation; the difference between the foreign gene and the host will affect the expression of the target protein; continuously repeated AT bases will end transcription in advance [35]. Therefore, codon optimization is a commonly adopted strategy for improved protein expression [36]. In this study, in order to further improve the expression level of the feIFN-ω in recombinant *P. pastoris*, the gene sequences encoding the feIFN-ω were subjected to optimization according to the codon usage bias for *P. pastoris*, such as using high-frequency dominant codon, reducing the GC content, adjusting AT-rich regions, etc. [37–39]. With the codon optimization, the expression level of the feIFN-ω’ (after optimization) by the recombinant GS115-pPICZαA-ω’ was nearly two times higher than that of the feIFN-ω (before optimization) expressed by the recombinant GS115-pPICZαA-ω, indicating that the optimization of codon usage bias for *P. pastoris* could significantly improve protein expression of the interest.

Subsequently, we systematically assessed the antiviral activity of the feIFN-ω’ *in vitro* and *in vivo. In vitro*, we first analyzed the antiviral activity of the supernatants secreted by GS115-pPICZαA-ω’, and results showed that the supernatants displayed effective antiviral activity with a dose-dependence, and significant reduction in CPE on the MDBK cells infected by VSV and FPV was observed, while not observed in negative control group. Then, we purified the recombinant feIFN-ω’ and further tested its antiviral activity, and our data showed that the purified feIFN-ω’ also displayed effective antiviral activity, and significant reduction in the FPV-induced CPE can be observed. By contrast, the antiviral effect of the feIFN-ω’ obtained in this study was better than that of INTERCAT IFN. Utilizing a standard VSV assay, we evaluated the antiviral effect of the feIFN-ω’ on F81 cells (feline), MDBK cells (bovine), and MDCK cells (canine) using the INTERCAT IFN as positive control, and our data showed that the feIFN-ω’ showed highly efficient antiviral effect against VSV on homologous and heterologous animal cells. However, the INTERCAT IFN only showed effective antiviral effect against VSV on homologous animal cells, but not on MDBK and MDCK, which was consistent with previous report [17].

Before performing *in vivo* experiments to evaluate the antiviral effect of the recombinant feIFN-ω’, in order to enhance the stability of the feIFN-ω’ and extend its half-life *in vivo*, the feIFN-ω’ was modified with polyethylene glycol. And then, the animal experiment was carried out. The cats were infected with feline parvovirus (FPV), followed by feIFN-ω’ treatment, using FPV-infected cats without interferon treatment as control. We found that the viral loads in blood, kidney, liver, spleen, intestine, and feces of the FPV-infected cats with the feIFN-ω’ treatment (feIFN-ω’ treatment group) significantly decreased, and the virus could be effectively eliminated detected by IHC assay, indicating that the FPV replication was effectively inhibited. However, the viral loads in FPV-infected cats without feIFN-ω’ treatment (FPV infection group) continued to increase. FPV infection can cause panleukopenia in cats. So, we determined the total WBC levels in the cats from mock control group, FPV infection group, and feIFN-ω’ treatment group. Our data showed that the total WBC counts significantly decreased after virus infection, and with the feIFN-ω’ treatment, the total WBC counts in feIFN-ω’ treatment group were gradually returned to normal, but not in FPV infection group, indicating effective therapeutic effect of the feIFN-ω’. To further explore the antiviral effect of the feIFN-ω’, the mRNA levels and protein levels of the antiviral proteins Mx1, ISG15, ISG56, and IFIT3 in the blood of the cats from each group were determined, and from these results, we clearly see that the feIFN-ω’ treatment can significantly promote the expression of antiviral proteins Mx1, ISG15, ISG56, and IFIT3, which would further exert their antiviral functions to inhibit the replication of FPV. Many studies have also demonstrated that interferon ω could stimulate the expression of the antiviral proteins ISGs [40,41]. In addition, we also found that the mRNA transcript levels of pro-inflammatory cytokines IL-1β, TNF-α, IL-6, and IL-12, and anti-inflammatory cytokines IL-4, and IL-10 tended to decrease (*p* < 0.05 or *p* < 0.01) in the cats from the feIFN-ω’ treatment group, compared with the FPV infection group. These findings are consistent with an overall reduction of pro-inflammatory pathways in cats treated with IFN [24].

**Figure 10.**
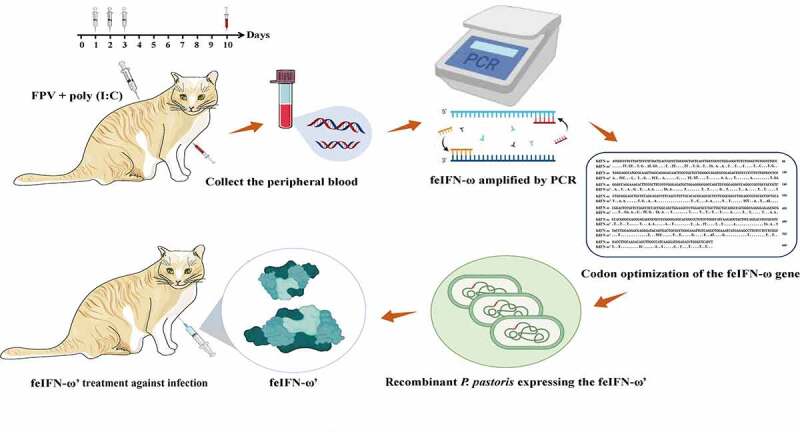
Global overview of the gene clone, codon optimization, expression, purification and antiviral effect evaluation of the novel feIFN-ω.

A global overview of the gene clone, codon optimization, expression, and antiviral effect evaluation of a novel feIFN-ω is given in Figure 10. In conclusion, we cloned a gene encoding a novel feIFN-ω, optimized its codons according to the codon usage bias of *P. pastoris*, and achieved highly efficient expression of the feIFN-ω from a recombinant *P. past*oris. The antiviral efficacy of the feIFN-ω was subsequently evaluated, and our results clearly demonstrated that the feIFN-ω displayed effective antiviral activity against VSV and FPV, suggesting a promising therapeutic agent for viral diseases in cats.

## Data Availability

The authors confirm that the data supporting the findings of this study are available within the article [and/or] its supplementary materials. https://www.ncbi.nlm.nih.gov/nuccore/MT754935.1
